# Systematic Degradation Rate Analysis of Surface-Functionalized Porous Silicon Nanoparticles

**DOI:** 10.3390/ma12040580

**Published:** 2019-02-15

**Authors:** Rae Hyung Kang, Seo Hyeon Lee, Sangrim Kang, Jinyoung Kang, Junho K. Hur, Dokyoung Kim

**Affiliations:** 1 Department of Biomedical Science, Graduate School, Kyung Hee University, Seoul 02447, Korea; hpohpo2000@hanmail.net (R.H.K.); lee19911230@gmail.com (S.H.L.); sangrimk@gmail.com (S.K.); 2 Department of Pathology, College of Medicine, Kyung Hee University, Seoul 02447, Korea; 3 Department of Chemistry and Biochemistry, University of California, San Diego, La Jolla, CA 92093, USA; j8kang@eng.ucsd.edu; 4 Department of Anatomy and Neurobiology, College of Medicine, Kyung Hee University, Seoul 02447, Korea; 5 Center for Converging Humanities, Kyung Hee University, Seoul 02447, Korea; 6 Biomedical Science Institute, Kyung Hee University, Seoul 02447, Korea

**Keywords:** porous silicon, nanoparticle, surface modification, silicon surface, degradation

## Abstract

Porous silicon nanoparticles (pSiNPs) have been utilized within a wide spectrum of biological studies, as well as in chemistry, chemical biology, and biomedical fields. Recently, pSiNPs have been constantly coming under the spotlight, mostly in biomedical applications, due to their advantages, such as controlled-release drug delivery in vivo by hydrolysis-induced degradation, self-reporting property through long life-time photoluminescence, high loading efficiency of substrate into pore, and the homing to specific cells/organ/bacteria by surface functionalization. However, the systematic degradation rate analysis of surface-functionalized pSiNPs in different biological media has not been conducted yet. In this paper, we prepared four different surface-functionalized pSiNPs samples and analyzed the degradation rate in six different media (DI H_2_O (deionized water), PBS (phosphate-buffered saline), HS (human serum), DMEM (Dulbecco’s modified Eagle’s medium), LB (lysogeny broth), and BHI (brain heart infusion)). The obtained results will now contribute to understanding the correlation between surface functionalization in the pSiNPs and the degradation rate in different biological media. The characterized data with the author’s suggestions will provide useful insights in designing the new pSiNPs formulation for biomedical applications.

## 1. Introduction

Porous silicon (pSi) is an inorganic silicon material that has nano-sized pores in its microstructure. Since its discovered in the mid-1950s, pSi has been applied within various research fields and industries. Its unique physical, chemical, and biological properties have been reported with interesting applications [1].

Generally, porous silicon can be prepared by the electrochemical etching method, using crystalline silicon wafer in hydrofluoric acid (HF) containing electrolytes [2,3]. In an electrochemical reaction with two electrodes, the silicon elements in the wafer are dissolved out into ionic forms, such as tetrafluorosilane (SiF_4_), hexafluorosilane (SiF_6_^2−^), and the resulting wafer have pores on the surface [3]. The pore diameter, porosity, and wall thickness can all be controlled by fabrication parameters; current density, wafer type (dopant type/density), composition of electrolyte, and others [4]. The generated porous silicon-containing wafer has been applied in the form of sensors in order to detect biohazard species, as well as disease biomarkers [5,6,7,8,9,10,11,12,13].

Recently, porous silicon has been applied in the biomedical research field, by generating porous silicon microparticles (pSiMPs) and nanoparticles (pSiNPs) [14,15,16,17,18,19,20,21,22,23]. The discovery of the quantum confinement effect and biodegradable property brings porous silicon into the spotlight [14,24,25,26,27]. In particular, pSiNPs display many advantages, including (i) high load efficiency toward substrates, such as drugs and peptides, (ii) superior controlled-release properties, (iii) no harmful byproduct generation after degradation, (iv) strong near-infrared (NIR) photoluminescence and two-photon (TP) absorbing ability for bio-imaging, (v) negligible cytotoxicity, and (vi) cell/organ/bacteria specific targeting abilities through fabrication of the surface.

The surface modification of pSiNPs is imperative in improving their properties and usage [3,28,29,30]. Freshly prepared pSiNPs have silicon hydroxide (Si–OH) functionality, primarily with minor silicon hydride (Si–H) and silicon oxide bridge (Si–O–Si). The silicon hydroxide moiety on the oxidized pSiNPs surface is a good platform for further surface modification for property enhancement; (i) hydrophobicity/hydrophilicity control in order to enhance the substrate loading efficacy and water solubility, (ii) controlled-release of the encapsulated substrate within the pore, (iii) the targeting of specific cell/organ/bacteria through the introduction of the homing moiety, such as peptides, ligands, and chemicals, and (v) tracking in vivo by introducing imaging agents, such as fluorophore.

To date, only a few surface modification methods for the surface of oxidized pSiNPs have been introduced into the field. The hydrolytic condensation with organo-silane reagents generates a new Si–O–Si bond through a reaction between Si–OH and (R_3_–Si–X, R = methoxy, ethoxy) on the surface of pSiNPs [14,20,29]. Most recently, the ring-opening click chemistry approach, based on 5-membered heterocyclic compounds containing a Si–S or Si–N bond within the ring was also reported [31,32]. This simple chemistry is, of course, well-known and widely practiced within bio-related works, including the (i) conjugation of biomolecules, such as protein, peptide, amino acid, and polymer, (ii) PEGylation (PEG: polyethylene glycol), and (iii) the controlled degradation of pSiNPs. However, there is no systematic analysis study result for the degradation rate of pSiNPs and their surface-functionalized products in different biological solutions including serum, cell culture media, and microorganism culture media.

In this study, we prepared four different types of pSiNPs samples and systematically analyzed their degradation rate in six different biological solutions. The main focus of this paper is to provide basic information on the effects between the surface functionalization of pSiNPs and their degradation rate under different environments. Researchers within these fields will now be able to find better surface functionalization routes catering to their purposes.

## 2. Materials and Methods

### 2.1. Materials

The chemical reagents were purchased from Creative PEG-Works (Chapel Hill, NC, USA), TCI (Tokyo, Japan), Nanocs (New York, NY, USA), and Samchun chemicals (Seoul, Korea). Commercially available reagents and anhydrous solvents were used without further purification. Chemical reaction and centrifugation were performed in an open-air environment at room temperature (25 °C). Silane-PEG-FITC (PEG: Polyethylene glycol, and FITC: Fluorescein isothiocyanate; M.W. = 5 kDa) (Product No. PG2-FCSL-5k, USA) and Maleimide-PEG-NHS (PEG: Polyethylene glycol, and NHS: N-hydroxysuccinimide; M.W. = 5 kDa) (Product No. PG2-MLNS-5k, USA) were purchased from Nanocs (USA). mPEG-silane (M.W. = 5 kDa) was purchased from Creative PEG-Works (Product No. PLS-2011, USA) [PEG: polyethylene glycol]. 3-Aminopropyl-dimethylethoxysilane (APDMES) was purchased from Fluorochem (Product No. S00750, Glossop, UK). (3-Mercaptopropyl) triethoxysilane (MPTES) was purchased from TCI (Product No. M1505, Japan). Ethanol (EtOH) was purchased from Samchun chemicals (Product No. E0223, Korea). Dulbecco’s modified eagle’s medium (DMEM, Product No. SH30243.01), Dulbecco’s phosphate buffered saline (DPBS, Product No. SH30028.02), and fetal bovine serum (FBS, SH30084.03) were purchased from Hyclone (Hamptom, NH, USA). Trypsin-EDTA (Product No. 25200-056) and Penicillin streptomycin (PS, Product No. 15140-122) were purchased from Gibco (Hamptom, NH, USA). Human serum was purchased from Sigma Aldrich (Product No. H4522, St. Louis, MO, USA). Luria-Bertani (LB, Product No. 244620) and brain heart infusion (BHI, product No. 211059) were purchased from BD Difco (Frankiln Lakes, NJ, USA). These media composition is listed in Supplementary Materials, Table S1.

### 2.2. Preparation of pSi Nanoparticles

Porous silicon nanoparticles (pSiNPs) were fabricated by electrochemical etching; constant current anodization of heavily boron-doped p-type single crystal silicon wafers (polished on the (100) face, Virginia Semiconductor, Inc. (Fredericksburg, VA, USA)) in aqueous ethanolic hydrofluoric acid electrolytes (Caution: HF is highly corrosive. Proper precautions are required when handling) [3]. The porous silicon films were prepared from silicon wafer by following “perforated etch” procedure [33]. The prepared porous silicon films (~40 mg) were fractured using ultrasonicator (VWR, Radnor, PA, USA) in deionized water (DI H_2_O, 4 mL) for 24 h and then filtered through a 0.22 μm syringe filter (Millipore, Millex syringe filter unit, 220 nm model #SLGP033RS). The pSiNPs were then further incubated in deionized water for 7 days at room temperature (25 °C) to form oxidized silicon surfaces (Si–OH). The resulting pSiNPs were collected using centrifugation (14,000 rpm, 15 min) and then washed 3 times with ethanol.

### 2.3. Study Design and Preparation of pSiNPs Samples

**Figure 1:**
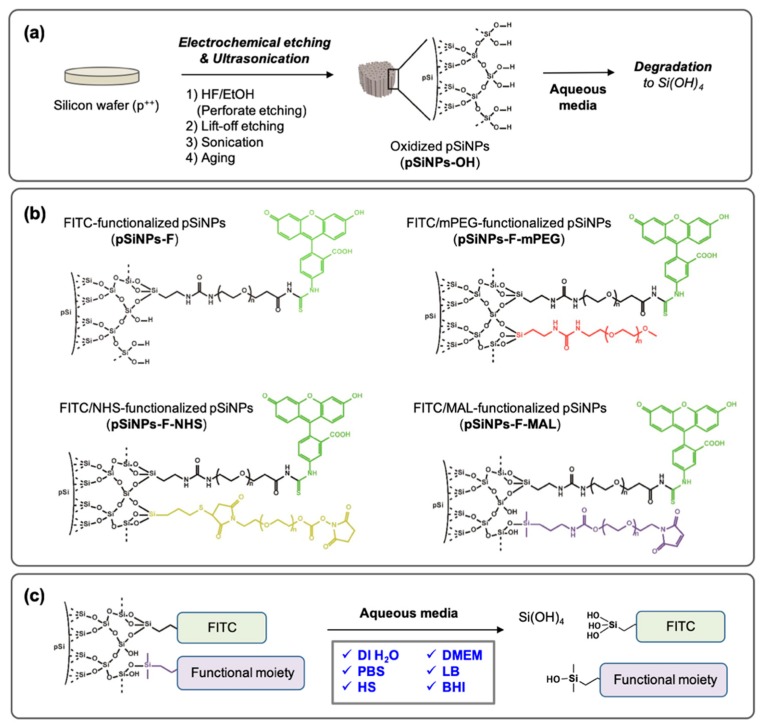
(**a**) Scheme for the preparation of oxidized porous silicon nanoparticles (pSiNPs). (**b**) Chemical structure of the pSiNPs samples used in this study. (**c**) Scheme for the degradation of the pSiNPs samples in aqueous media.

The pSiNPs could be prepared by following the known electrochemical etching methods in ethanolic HF solution. The perforate etching method generated nanoparticles even in size. The lift-off etching in low HF concentration solution and ultrasonic fracture in deionized water (DI H_2_O) gave as-prepared pSiNPs, and was followed by an aging-step (dispersion in DI H_2_O for days/weeks at room temperature (25 °C) to generate hydroxide functionality (Si–OH) on the surface, Figure 1a). Generally, the degradation of oxidized pSiNPs, named **pSiNPs-OH**, occurs in aqueous media and orthosilicic acid (Si(OH_4_) is generated (Figure 1a). The degradation rate of **pSiNPs-OH** depends on the solution (pH, ion concentration, chemical, etc.), temperature, external stimulus (light, sonication, etc.), particle size, pore size, particle concentration, and surface functionality. To analyze only the effect of surface functionality, we designed four different oxidized pSiNPs formulations (Figure 1b): (i) FITC (fluorescein isothiocyanate)-tagged pSiNPs via PEG linker (5 kDa), named **pSiNPs-F**. The degradation of pSiNPs release the FITC-PEG out into the solution, thus the following of FITC fluorescence signal allowed us to analyze the degradation rate (Figure 1c). (ii) mPEG (5 kDa)-functionalized **pSiNPs-F**, named **pSiNPs-F-mPEG**. PEGylation with terminal methoxy-PEG is widely used to enhance biocompatibility as well as the EPR (enhanced permeability and retention) effect at the disease site, especially in cancer-specific drug delivery systems. (iii) NHS (N-hydroxysuccinimide)-functionalized **pSiNPs-F**, named **pSiNPs-F-NHS**. NHS is an amine-reactive moiety; therefore, it is widely used to conjugate chemicals or peptides that have a primary amine (-NH_2_) group [34,35]. (iv) MAL (maleimide)-functionalized **pSiNPs-F**, named **pSiNPs-F-MAL**. MAL is a thiol-reactive moiety; thus, it is widely used to conjugate peptide or protein that has thiol (–SH) group [34,35,36]. The surface functional moiety was introduced by hydrolytic condensation in an ethanol solution reaction between trialkoxysilane and hydroxylated silicon surface (pSi-OH + Si(OR)_3_-X → pSi-O-Si(OR)_2_-X + ROH, R = alkyl, X = functional PEG).

Next, we chose six different biological solutions to analyze the degradation rate of the pSiNPs samples (Figure 1c): (i) deionized water (DI H_2_O), (ii) phosphate-buffered saline (PBS), (iii) Dulbecco’s modified Eagle’s medium (DMEM); cell culture media, (iv) human serum (HS), (v) lysogeny broth (LB); bacteria (*E. coli*, *S. aureus*, etc.) culture media, and (vi) brain heart infusion (BHI); microorganism (bacteria, yeasts, molds, etc.) culture media. The degradation rate of each pSiNPs sample was analyzed by monitoring the FITC signal, which collected in the supernatant of each solution at given time-points (0–120 min).

### 2.4. Surface Modification of pSi Nanoparticles

#### 2.4.1. Fabrication of pSiNPs Surface with Triethoxysilane-PEG-Fluorescein Isothiocyanate; Named **pSiNPs-F**

The oxidized pSiNPs (**pSiNPs-OH**) were collected from stock dispersion within deionized water through centrifugation (14,000 rpm, 15 min, 3 times). The centrifuged pSiNPs pellet (~1 mg) was re-dispersed in ethanol (800 μL), triethoxysilane-PEG-FITC (5 kDa) stock solution (5 mg/mL, 200 μL) were added, and then mixed using a vortex mixer at room temperature (25 °C) for 2 h. The resulting particles were washed 3 times with ethanol by using centrifugation (14,000 rpm, 15 min) to remove the remaining triethoxysilane-PEG-FITC.

#### 2.4.2. Fabrication of pSiNPs-F with mPEG (Triethoxysilane-PEG-Monomethoxy); Named **pSiNPs-F-mPEG**

The pSiNPs-F nanoparticle (~1 mg) and triethoxysilane-PEG-monomethoxy (5 kDa) stock solution (5 mg/mL, 200 μL) were dispersed in ethanol (800 μL), and then mixed using a vortex mixer at room temperature (25 °C) for 2 h. The resulting particles were washed 3 times with ethanol by using centrifugation (14,000 rpm, 15 min) to remove the remaining triethoxysilane-PEG-monomethoxy.

#### 2.4.3. Fabrication of pSiNPs-F with (3-Mercaptopropyl)Triethoxysilane (MPTES) and Maleimide (MAL)-PEG-N-Hydroxysuccinimide (NHS); Named **pSiNPs-F-NHS**

The pSiNPs-F nanoparticle (~1 mg) and (3-mercaptopropyl)triethoxysilane (MPTES, 20 μL) were dispersed in ethanol (1 mL), and then mixed using a vortex mixer in room temperature (25 °C) for 2 h. The thiolated nanoparticles were then washed 3 times with ethanol by using centrifugation (14,000 rpm, 15 min) to remove the remaining MPTES. The thiolated nanoparticles were resuspended in ethanol (800 μL), and maleimide-PEG-NHS stock solution (5 mg/mL, 200 μL) was added. The mixture was vortexed at room temperature (25 °C) for 2 h. The resulting particles were washed 3 times with ethanol by using centrifugation (14,000 rpm, 15 min) to remove the remaining MAL-PEG-NHS.

#### 2.4.4. Fabrication of pSiNPs-F with 3-Aminopropyl-Dimethylethoxysilane (APDMES) and Maleimide (MAL)-PEG-N-Hydroxysuccinimide (NHS); Named **pSiNPs-F-MAL**

The pSiNPs-F nanoparticle (~1 mg) and 3-aminopropyl-dimethylethoxysilane (APDMES, 20 μL) were dispersed in ethanol (1 mL), and then mixed using a vortex mixer at room temperature (25 °C) for 2 h. The aminated nanoparticles were then washed 3 times with ethanol by using centrifugation (14,000 rpm, 15 min) to remove the remaining APDMES. The aminated pSiNPs were resuspended in ethanol (800 μL), and MAL-PEG-NHS stock solution (5 mg/mL, 200 μL) was added. The mixture was vortexed at room temperature (25 °C) for 2 h. The resulting particles were washed 3 times with ethanol by using centrifugation (14,000 rpm, 15 min) to remove the remaining MAL-PEG-NHS.

### 2.5. Characterization of Nanoparticles

Dynamic light scattering (DLS) and zeta-potential of the pSiNPs samples (**pSiNPs-OH**, **pSiNPs-F**, **pSiNPs-F-mPEG**, **pSiNPs-F-NHS**, and **pSiNPs-F-MAL**) were measured by Malvern Instruments Zetasizer Nano ZS90 (Worcester-shire, UK). The morphologies of nanoparticles were characterized by transmission electron microscopy (Tecnai, G2 F30ST, FEI Company, Hillsboro, OR, USA). Attenuated total reflectance Fourier transform infrared (ATR-FTIR, Thermo Fisher Scientific, Waltham, MA, USA) was used to observe the surface functional group of each nanoparticle.

### 2.6. Spectroscopic Study

UV/Vis absorption spectra were obtained using spectro-photometer (Agilent Technologies, Santa Clara, CA, USA). Fluorescence spectra were measured with a spectro-fluorophotometer (Shimadzu Corp., Kyoto, Japan), with a 1 cm standard quartz cell (internal volume of 0.1 mL, Hellma Analytics, Jena, Germany). Fluorescence intensity variations were analyzed in deionized water (DI H_2_O), PBS, human serum (HS), DMEM, LB, and BHI at 37 °C for 0–120 min.

### 2.7. Cytotoxicity

The cytotoxicity of the pSiNPs samples against HeLa cells was evaluated using the 3-(4,5-dimethylthiazol-2-yl)-2,5-diphenyltetrazolium bromide (MTT) assay according to the manufacturer’s instructions. The cells (5 × 10^4^ per well) were seeded in 96-well plates and incubated for 24 h at 37 °C in a humidified 5% CO_2_ incubator. Afterward, the cells were treated with 25, 50, 100, and 200 μg/mL concentrations of the nanoparticles and the cell toxicity was measured after a 2 h incubation. Later, 10 μL of MTT solution (5 mg/mL) in PBS was added to each well of a 96-well plate, followed by incubation for 4 h at 37 °C. The Formazan that formed at the end of the reaction was dissolved in 150 μL of dimethyl sulfoxide (DMSO) and the absorbance was measured at a wavelength of 570 nm using a microplate reader (Multiskan FC, Thermo Fisher, Waltham, MA, USA). The percentage of cell cytotoxicity was calculated using the formula; Cell viability(%) = (Mean OD of sample × 100)/(Mean OD of the control group) (OD: optical density).

## 3. Results and Discussion

### 3.1. Characterization of the pSiNPs Samples

**Figure 2:**
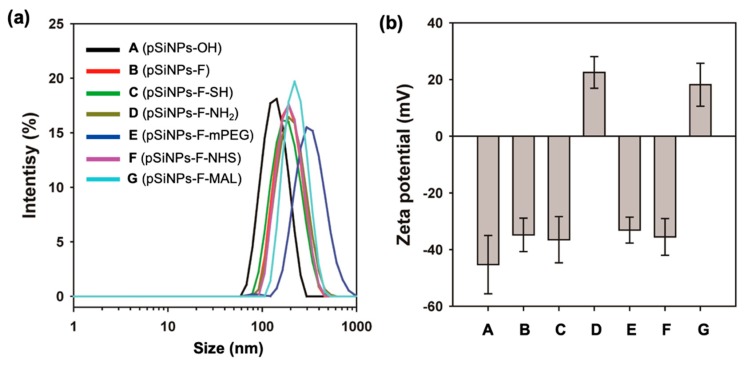
(**a**) Mean hydrodynamic diameter (intensity distribution) measured by dynamic light scattering (DLS), and (**b**) zeta-potential value of pSiNPs and its surface-functionalized samples. Each mean and standard deviation was calculated in triplicate.

First, we characterized each pSiNPs sample. As-prepared pSiNPs (**pSiNPs-OH**) displayed average hydrodynamic diameters of ~140 nm and −45.3 ± 10.3 mV, within the DLS (dynamic light scattering) analysis and zeta-potential measurement (Figure 2, Table 1). Reaction of the **pSiNPs-OH** with triethoxysilane-PEG-FITC (5 kDa, named silane-PEG-FITC) proceeded to completion within 2 h, and resulted with the **pSiNPs-F** showing a slight increase in size (~190 nm) with a decreased surface charge (−34.8 ± 5.91 mV), due to the Si–O–Si bond formation via the hydrolytic condensation between silicon hydroxide and triethoxysilane-PEG-FITC. The introduction of methoxy-terminal triethoxysilane-PEG (5 kDa, named mPEG) on the **pSiNPs-OH** gave a size increase up to ~350 nm, but no significant surface charge change (−33.1 ± 4.56 mV). The NHS-/MAL-terminal pSiNPs samples were prepared using the amine-/thiol-terminal intermediate. The surface of **pSiNPs-F** was fabricated using 3-aminopropyl-dimethyl-ethoxysilane (APDMES) and (3-mercaptopropyl) triethoxysilane (MPTES), respectively, through hydrolytic condensation. The reaction product showed no significant size change, but a dramatic zeta potential change for APDMES, due to the positive primary amine moiety (22.5 ± 5.61 mV). The amine-/thiol-terminal **pSiNPs-F** intermediate was further modified to **pSiNPS-F-NHS** and **pSiNPs-F-MAL** using difunctional PEG (5 kDa, one terminal maleimide, one terminal N-hydroxysuccinimide) via thiol-ene addition (for **pSiNPs-F-NHS**) and amide formation (for **pSiNPs-F-MAL**). The resulting samples showed a slight increase in size (~210 nm for **pSiNPs-F-NHS**, ~240 nm for **pSiNPs-F-MAL**). The **pSiNPs-F-MAL** showed a positive zeta-potential (18.2 ± 7.57 mV), due to the remaining amine moiety on the surface.

**Figure 3:**
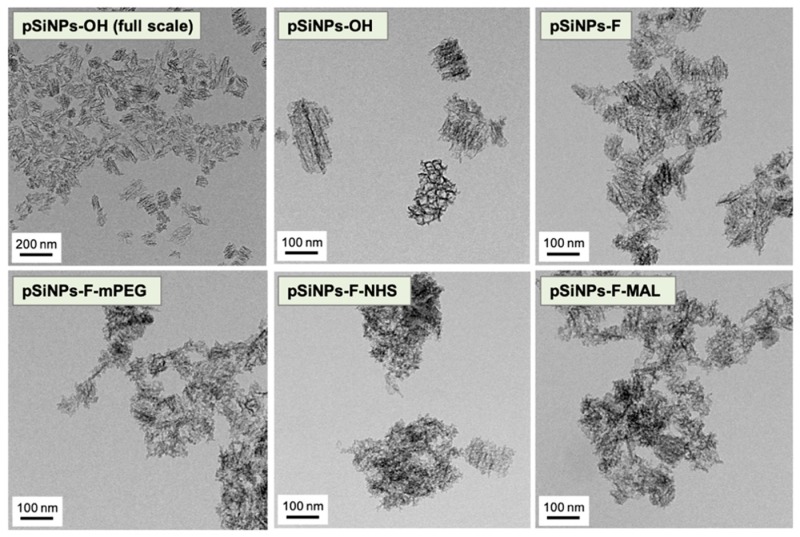
Transmission electron microscopy (TEM) images of oxidized pSiNPs (**pSiNPs-OH**) and its surface-functionalized samples. Scale bar is indicated in each figure.

Transmission electron microscopy (TEM, FEI company, Hillsboro, OR, USA) images indicated that the particle size is homogenous and the open pore structure of the **pSiNPs-OH** was preserved with no significant pore wall collapse (Figure 3). The other pSiNPs samples also maintained the porous structure after the surface functionalization. The aggregation-like structure was induced in the TEM sampling step, not an actual aggregation within the aqueous solution (see Table 1 for DLS and PDI (Poly dispersity index)).

**Figure 4:**
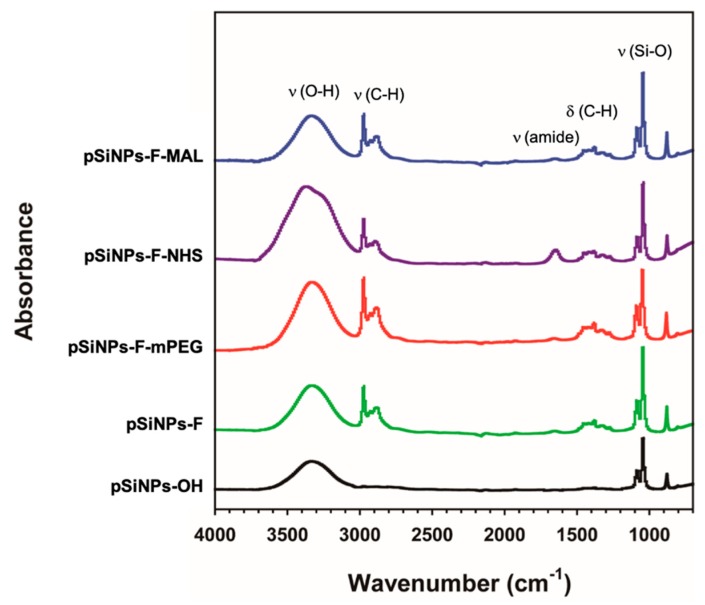
Attenuated total reflectance Fourier-transform infrared (ATR-FTIR) spectra of the **pSiNPs-OH** and its surface-functionalized samples. Symbols: υ = stretching, δ = bending, Si = peaks associated with porous silicon surface.

The attenuated total reflectance Fourier-transform infrared (ATR-FTIR, Thermo Fisher Scientific, Waltham, MA, USA) spectrum of the pSiNPs samples was monitored (Figure 4). The infrared spectrum of the pSiNPs-OH displayed bands associated with the Si–O–H functionality; broad band for υ(O–H) at 3550–3200 cm^−1^, υ(Si–O) at 1065 nm^−1^ (black trace) [3,30,31]. The other pSiNPs samples showed strong bands at 2950–2850 cm^−1^, 1480–1350 cm^−1^, and 1360–1080 cm^−1^ associated with υ(C–H), δ(C–H), and υ(amide bond), respectively, which derived from the PEG linker. The bending mode of amide bond dramatically appeared at 1650 cm^−1^ (purple trace). The peaks below 1000 cm^−1^ could be assigned to modes associated with bending SiH and SiH_2_.

**Figure 5:**
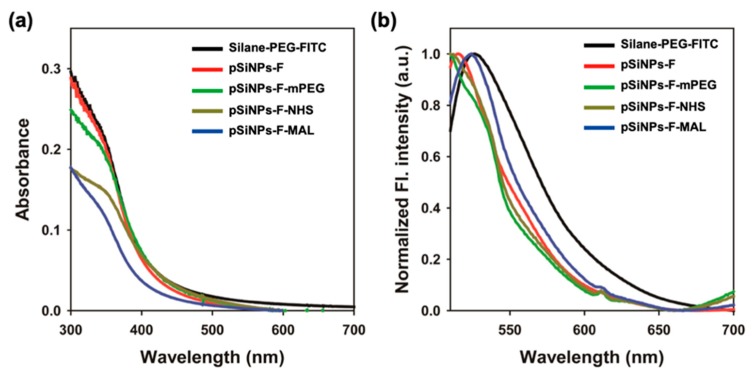
(**a**) UV/vis absorption and (**b**) normalized fluorescence spectra of triethoxysilane -PEG-FITC (black trace, Silane-PEG-FITC, 100 μg/mL) and pSiNPs samples in DI H_2_O (1.0 mg/mL). The fluorescence spectra were recorded after excitation at 495 nm at room temperature (25 °C).

Next, we monitored the fluorescence property of the pSiNPs samples (Figure 5). The surface grafting reagent triethoxysilane-PEG-FITC (Silane-PEG-FITC) showed a strong absorbance within the UV/Vis regions. Typically, FITC itself gives an absorption band at a visible region between 430 and 540 nm, but Silane-PEG-FITC showed major absorbance at the UV region between 300 and 500 nm (Figure 5a). All the FITC-PEG fabricated pSiNPs samples gave similar results, which was probably caused by the absorption of the PEG backbone. In the fluorescence spectra, all the samples showed strong FITC fluorescence spectra between 510 and 600 nm upon excitation at 495 nm (Figure 5b). From this data, we confirmed that the FITC fluorophore seemed to be located far-off from the pSiNPs surface; thus, the fluorescence quenching effect of silicon to fluorophore is negligible and the detached FITC-PEG side-product maintained their own fluorescence.

### 3.2. Degradation Rate Study for the pSiNPs Samples in Different Solutions

**Figure 6:**
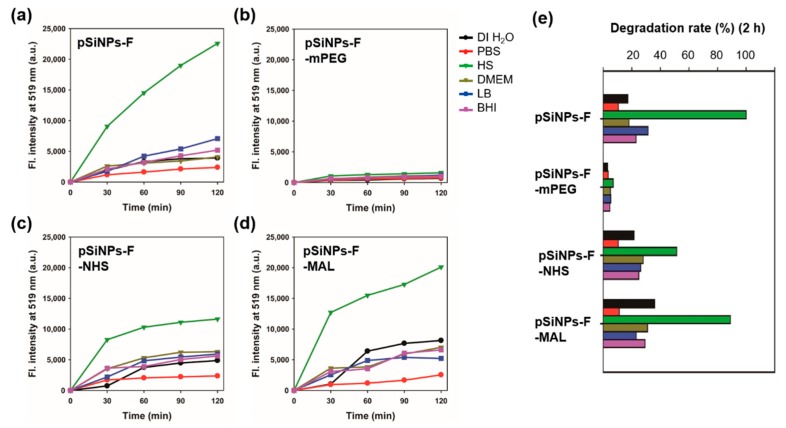
(**a**–**d**) Time-dependent fluorescence intensity (at 519 nm) plot of supernatant for the pSiNPs samples (100 μg/mL) in different biological solutions incubated at 37 °C for 0–120 min; black = DI H_2_O, red = PBS, olive = HS, khaki = DMEM, blue = LB, and purple = BHI. The supernatant was collected at the given time point (30 min intervals) using centrifugation (14,000 rpm, 15 min) of the pSiNPs-containing solution. The fluorescence intensity was corrected based on the fluorescence intensity of Silane-PEG-FITC in each solution (Figures S1 and S2 in Supplementary Materials). The fluorescence intensity was recorded after excitation at 495 nm at room temperature (25 °C). (**e**) Degradation rate (%) plot of the pSiNPs samples under given conditions.

With the basic characterization data of the pSiNPs samples, we analyzed their degradation rate in different six biological solutions (DI H_2_O, PBS, HS, DMEM, LB, and BHI) (Figure 6, Table 2). We added pSiNPs sample (100 μg/mL) into solutions and then incubated them at 37 °C for 0–120 min. Every 30 min, we collected the supernatant of each solution and measured the fluorescence signal from the silane-PEG-FITC that peeled off from the surface of pSiNPs by degradation (Figure 1c). The degradation rate results of each pSiNPs sample in different solutions are summarized as below (see the media composition in Table S1, Supplementary Materials).

- *DI H_2_O*: Maleimide group terminal **pSiNPs-F-MAL** gave a faster degradation rate (half-degradation time-point at 166 min, see Table 2) than the other pSiNPs samples. The methoxy group terminal **pSiNPs-F-mPEG** showed a slower degradation rate that indicated a high stability.
- *PBS*: Most of the pSiNPs samples showed a slow degradation rate (half-degradation time-point at around 533–1713 min). Similar to DI H_2_O, **pSiNPs-F-mPEG,** that gave a high stability in PBS media.
- *HS*: In HS media, most of pSiNPs showed a fast degradation rate (half-degradation time-point at 60 min for **pSiNPs-F**, 116 min for **pSiNPs-F-NHS**, 67 min for **pSiNPs-F-MAL**) except **pSiNPs-F-mPEG** (850 min). The proteins, electrolytes, antibodies, and antigens in HS media seemed to accelerate the hydrolysis of the oxidized silicon surface, through approaching the non-functionalized area of **pSiNPs-F** and conjugation-induced enhanced hydrolysis (**pSiNPs-F-NHS**, **pSiNPs-F-MAL**). The methoxy-PEG functionalized **pSiNPs-F-mPEG** showed high stability even in HS media.
- *DMEM*: All the pSiNPs samples showed a moderate degradation rate (half-degradation time-point at 193–1190 min) in DMEM media. However, this data indicates that the degradation of surface-functionalized **pSiNPs-F-NHS** and **pSiNPs-F-MAL** became more significant in cell growth media after approximately 200 min. The high concentration of amine and glucose component in DMEM works as Lewis base, and, thus, the degradation was accelerated (aqueous oxidation induced by cationic surfactants).
- *LB*: Similar to DMEM, the pSiNPs samples showed a moderate degradation rate (half-degradation time-point at 192–1135 min) in LB. Yeast extract and tryptone do not appear to have crucial roles in the surface hydrolysis of pSiNPs.
- *BHI*: Similar to DMEM, the pSiNPs samples showed a moderate degradation rate (half-degradation time-point at 205–1310 min) in BHI. Calf brain infusion/beef heart infusion, proteose peptone, and disodium phosphate do not appear to have definite roles in the surface hydrolysis of pSiNPs.

We came to the following conclusions which will help researchers who use surface-functionalized pSiNPs samples in biological studies: (i) Non-functional PEG groups can enhance the stability of pSiNPs in most biological media. (ii) In HS, simple pSiNPs and NHS-/MAL-functionalized pSiNPs showed significantly fast degradation rates, thus they need to have further surface chemistry or conjugation before in vivo/in vitro treatments (blood vessel injection, incubation in HS, etc.). (iii) In PBS, most of pSiNPs seemed to be stable under the given condition; thus, the preparation of pSiNPs stock solution in PBS should be done before in vivo/in vitro treatments. (iv) The NHS-/MAL-functionalized pSiNPs samples showed a moderate degradation rate (below 30% within 2 h incubation) in LB and BHI, and, thus, this kind of formulation could be applied for the study related to bacteria or microorganism.

### 3.3. Cell Viability Assay of the pSiNPs Samples

**Figure 7:**
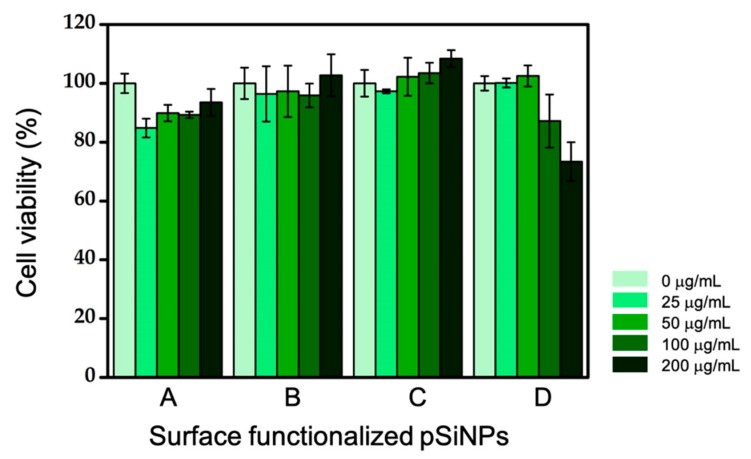
Effect of surface-functionalized pSiNPs samples on the cytotoxicity of HeLa cells. A: **pSiNPs-F**, B: **pSiNPs-F-mPEG**, C: **pSiNPs-F-NHS**, D: **pSiNPs-F-MAL**. HeLa cells were incubated with the pSiNPs samples (0–200 μg/mL) for 2 h. Means and standard deviation was calculated in triplicate. To avoid absorption band overlap between chemicals in MTT kit and FITC, we recorded the absorption at 570 nm.

In order to show the potential of each pSiNPs sample for biological study, we treated them in the HeLa cell (immortalized human cervical cancer cell) and measured their cell viability using the MTT method (Figure 7). The cell viability (%) value at 200 μg/mL was calculated by comparing the optical density (OD) with that of the control cells whose viability was taken as 100%. As shown in Figure 7, the viability of the cells with three pSiNPs samples including **pSiNPs-F**, **pSiNPs-F-mPEG**, and **pSiNPs-F-NHS** were more than 90%, at the treated concentration (0–200 μg/mL). On the other hand, the case of the **pSiNPs-F-MAL** decreased slightly to about 73% in comparison to the cell viability of control at a concentration of 200 μg/mL. We also observed cell morphology by the microscopic images of the control and the treated group, with different concentrations of the pSiNPs samples (data not shown). The treatment of the pSiNPs samples showed no abrupt effect on the cells, and also there were no visible morphological changes, such as rounding or shrinking. These results show that the surface-functionalized pSiNPs samples had no significant toxicity at the given concentrations and generated no negative effects.

## 4. Conclusions

We have shown the systematic degradation rate analysis of four different surface-functionalized pSiNPs in six different biological media. We prepared oxidized pSiNPs with Si–OH functionality, and functionalized its surface with FITC-conjugated silane PEG via hydrolytic condensation, in order to simply monitor the degradation rate of pSiNPs. By using FITC-PEG-functionalized pSiNPs, we prepared three different pSiNPs samples with methoxy-terminal PEG, NHS-terminal PEG, and MAL-terminal PEG, respectively. We fully characterized the particle properties using DLS/PDI analysis, zeta-potential measurement, TEM images, and ATR-FTIR analysis.

Within the degradation rate analysis, most of the pSiNPs samples showed a fast degradation rate, ~100% degradation before 2 h incubation at 37 °C in HS media, except methoxy-PEG-silane functionalized pSiNPs, **pSiNPs-F-mPEG**. The **pSiNPs-F-mPEG** showed a high stability within all the biological media, including DI H_2_O, PBS, HS, DMEM, LB, and BHI, within the given conditions. Amine-/thiol-group reactive moiety NHS-/MAL-terminal pSiNPs samples showed a moderate degradation rate (half-degradation time point at 166–574 min) in most of the solutions except HS. The characterization data of the pSiNPs samples along with the author’s suggestions will provide useful insights for the design of new pSiNPs formulations in accordance with experiment goals. In addition, the described results within this paper will attract great interests for further investigation, especially in fields related to silicon surface modification, and provide opportunities for further exploitation within the biomedical applications.

## Figures and Tables

**Table 1:** DLS/PDI and Zeta-potential of the pSiNPs samples.^1^ PDI: poly dispersity index.

Media	pSiNPs -OH	pSiNPs -F	pSiNPs -F-SH	pSiNPs -F-NH2	pSiNPs -F-mPEG	pSiNPs -F-NHS	pSiNPs -F-MAL
DLS	138.3	189.8	177.3	200.1	348.2	208.4	240.7
PDI	0.15	0.07	0.12	0.15	0.23	0.21	0.24
Zeta	−45.3 ± 10.3	−34.8 ± 5.91	−36.5 ± 8.16	22.5 ± 5.61	−33.1 ± 4.56	−35.5 ± 6.48	18.2 ± 7.57

^1^ DLS/PDI and zeta-potential values are measured in DI H_2_O. Each mean and standard deviation was calculated in triplicate.

**Table 2:** Calculated half-degradation time-points for the pSiNPs samples.^1^ Unit: min.

Media	pSiNP-F	pSiNPs-F-mPEG	pSiNPs-F-NHS	pSiNPs-F-MAL
DI H_2_O	346 (5.8 h)	1965 (32.8 h)	278 (4.6 h)	166 (2.8 h)
PBS	567 (9.5 h)	1713 (28.5 h)	574 (9.6 h)	533 (8.9 h)
HS	60 (1.0 h)	850 (14.2 h)	116 (1.9 h)	67 (1.1 h)
DMEM	332 (5.5 h)	1190 (19.8 h)	215 (3.6 h)	193 (3.2 h)
LB	192 (3.2 h)	1135 (18.9 h)	228 (3.8 h)	260 (4.3 h)
BHI	261 (4.4 h)	1310 (21.8 h)	241 (4.0 h)	205 (3.4 h)

^1^ The values calculated from the fluorescence intensity plot under given conditions; nanoparticle concentration (100 μg/mL), temperature (37 °C), particle size (naked **pSiNPs-OH**: 138.3 nm), and media composition.

## Supplementary Materials

The following is available online at http://www.mdpi.com/1996-1944/12/4/580/s1, Table S1: media composition; Figure S1: (a) UV/Vis absorption and (b) fluorescence spectra (λ_ex_: 495 nm) of triethoxysilane-PEG-FITC (100 μg/mL) in deionized water (DI H_2_O), phosphate-buffered saline (PBS), human serum (HS), Dulbecco’s modified Eagle medium (DMEM), lysogeny broth (LB), and brain heart infusion (BHI) at 25 °C; Figure S2: Time-dependent fluorescence spectra (λ_ex_: 495 nm) of the supernatant from pSiNPs samples (100 μg/mL) in deionized water (DI H_2_O), phosphate-buffered saline (PBS), human serum (HS), Dulbecco’s modified Eagle medium (DMEM), lysogeny broth (LB), and brain heart infusion (BHI) at 25 °C for 0–120 min. The supernatant was collected after the centrifugation (15,000 rpm, 15 min) at a given time point.
