# Supplementary material for: Systematic Degradation Rate Analysis of Surface-Functionalized Porous Silicon Nanoparticles
Source: Materials (Basel). 2019 Feb 15;12(4):580. doi: 10.3390/ma12040580 (PMC6416615; doi:10.3390/ma12040580)
Supplement: Supplementary file 1 [file materials-12-00580-s001.pdf]

Supplementary Materials: Systematic Degradation Rate Analysis of Surface-functionalized Porous Silicon Nanoparticles

Rae Hyung Kang [1,†], Seo Hyeon Lee [1,†], Sangrim Kang [1,2,†], Jinyoung Kang [3], Junho K Hur [2,*] and Dokyoung Kim [1,4,5,6,*]

Table S1. Media composition. Abbreviation: DMEM; Dulbecco's modified Eagle medium, LB; lysogeny broth, and BHI; brain heart infusion.

Media	DMEM	LB	BHI
Composition	4.0 mM L-Glutamine<br>4500 mg/L Glucose<br>Sodium Pyruvate	Tryptone<br>Yeast Extract<br>Sodium Chloride	Calf Brain, Infusion<br>Beef Heart, Infusion<br>Proteose peptone<br>Sodium Chloride<br>Disodium Phosphate
pH	7.4	7.0 ± 0.2	7.4 ± 0.2

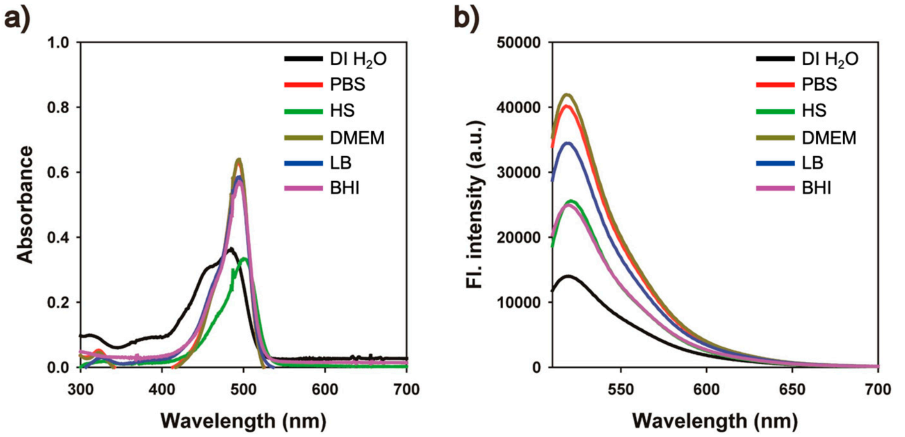

Figure S1. (**a**) UV/Vis absorption and (**b**) fluorescence spectra (λ_{ex}: 495 nm) of triethoxysilane-PEG-FITC (100 µg/mL) in deionized water (DI H_2O), phosphate-buffered saline (PBS), human serum (HS), Dulbecco's modified Eagle medium (DMEM), lysogeny broth (LB), and brain heart infusion (BHI) at 25 °C.

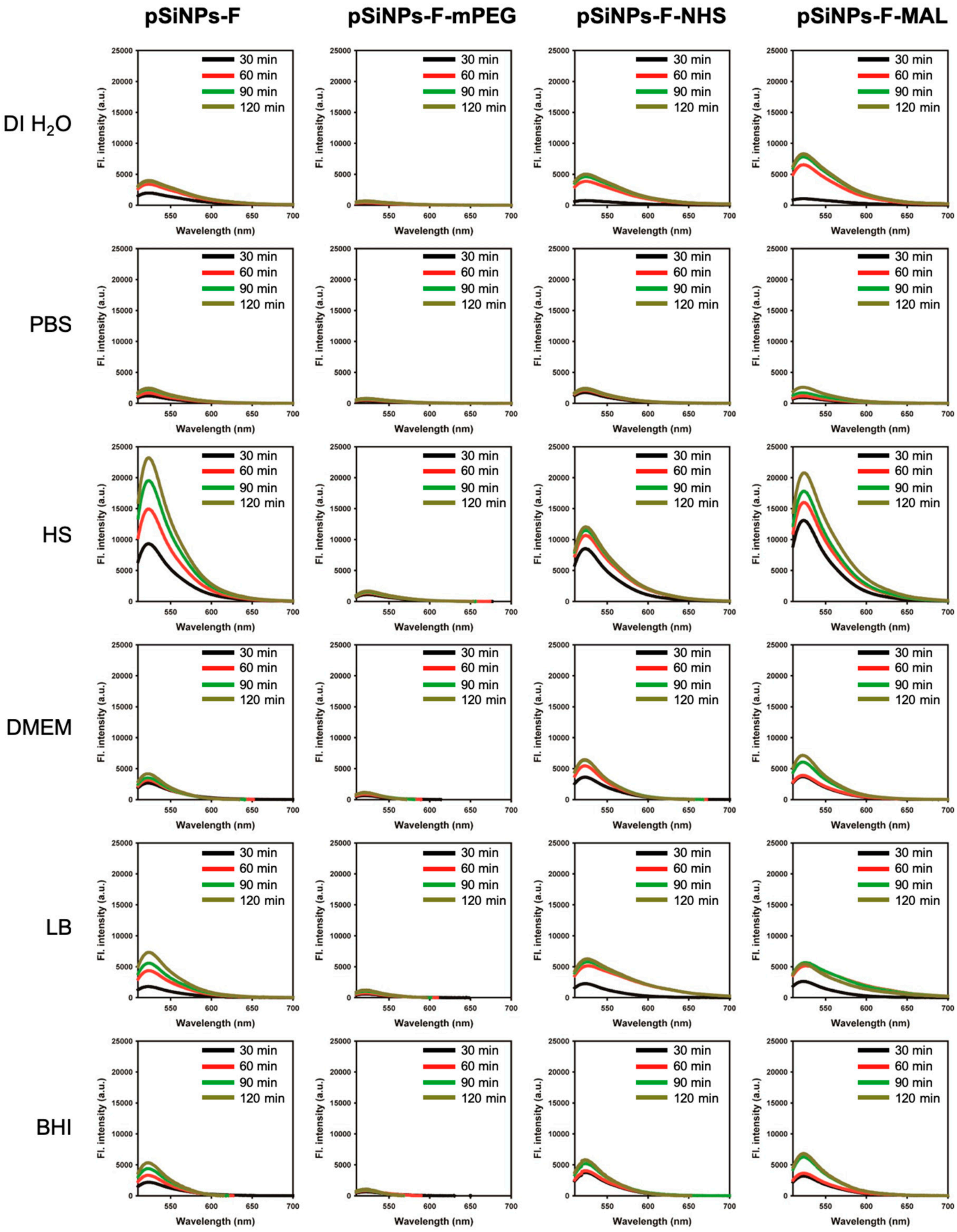

Figure S2. Time-dependent fluorescence spectra (λ_{ex}: 495 nm) of the supernatant from pSiNPs samples (100 μg/mL) in deionized water (DI H_2O), phosphate-buffered saline (PBS), human serum (HS), Dulbecco's modified Eagle medium (DMEM), lysogeny broth (LB), and brain heart infusion (BHI) at 25 °C for 0–120 min. The supernatant was collected after the centrifugation (15,000 rpm, 15 min) at given time point.
